# Observer Performance in the Use of Digital and Optical Microscopy for the Interpretation of Tissue-Based Biomarkers

**DOI:** 10.1155/2014/157308

**Published:** 2014-11-11

**Authors:** Marios A. Gavrielides, Catherine Conway, Neil O'Flaherty, Brandon D. Gallas, Stephen M. Hewitt

**Affiliations:** ^1^Division of Imaging, Diagnostics, and Software Reliability, Office of Science and Engineering Laboratories, Center for Devices and Radiological Health, U.S. Food and Drug Administration, Silver Spring, MD 20993, USA; ^2^Laboratory of Pathology, Center for Cancer Research, National Cancer Institute, National Institutes of Health, Bethesda, MD 20892, USA; ^3^Leica Biosystems, Vista, CA 92081, USA

## Abstract

*Background.* We conducted a validation study of digital pathology for the quantitative assessment of tissue-based biomarkers with immunohistochemistry. *Objective.* To examine observer agreement as a function of viewing modality (digital versus optical microscopy), whole slide versus tissue microarray (TMA) review, biomarker type (HER2 incorporating membranous staining and Ki-67 with nuclear staining), and data type (continuous and categorical). *Methods.* Eight pathologists reviewed 50 breast cancer whole slides (25 stained with HER2 and 25 with Ki-67) and 2 TMAs (1 stained with HER2, 1 with Ki-67, each containing 97 cores), using digital and optical microscopy. *Results.* Results showed relatively high overall interobserver and intermodality agreement, with different patterns specific to biomarker type. For HER2, there was better interobserver agreement for optical compared to digital microscopy for whole slides as well as better interobserver and intermodality agreement for TMAs. For Ki-67, those patterns were not observed. *Conclusions.* The differences in agreement patterns when examining different biomarkers and different scoring methods and reviewing whole slides compared to TMA stress the need for validation studies focused on specific pathology tasks to eliminate sources of variability that might dilute findings. The statistical uncertainty observed in our analyses calls for adequate sampling for each individual task rather than pooling cases.

## 1. Introduction

Digital pathology (DP) is an image-based environment that enables the acquisition, management, and interpretation of pathology information generated from whole slide images (WSI). The potential advantages of DP include telepathology, digital consultation and slide sharing, pathology education, indexing and retrieval of cases, and the use of automated image analysis [1–4]. Digital pathology was enabled by recent technological advances in WSI systems, which can digitize microscope slides at high resolution in an automated manner. The FDA has determined that DP is subject to regulatory oversight. Validation studies can identify possible limitations for specified intended use of DP and provide necessary information for regulatory approval of DP devices for that intended use. Recent guidelines for validation of WSI from the College of American Pathologists state that “validation is recommended to determine that a pathologist can use a WSI system to render an accurate diagnosis with the same or better level of ease as with a traditional microscope and without interfering artifacts or technological risks to patient safety” [5].

Validation studies can be further categorized into two main assessment types. (1) Technical or objective assessment of WSI systems: this assessment aims to characterize the technical performance of the components in the imaging chain, including the light source, optics, and sensor for image acquisition, as well as embedded algorithm systems for autofocusing, selecting, and combining different fields-of-view (FOV) in a composite image, image compression, and color correction. (2) Observer-based assessment: this assessment, in the absence of a reference standard, is typically performed as a comparison of inter- and intraobserver agreements between digital and optical microscopy for specific pathology tasks. In this study we only focus on the observer-based assessment of WSI systems.

A number of validation studies have been reported for* diagnostic* tasks in a number of applications [6–15]. Our study focused more specifically on the validation of DP for the observer-based* immunohistochemical* (IHC) assessment of tissue-based biomarkers, that is, the manual review of IHC without the use of image analysis. Even though a large number of studies have reported on software applications for quantitative IHC on WSI images, both in the research [16–26] and in commercial environment [27–35], only a small number of* validation* studies have focused on comparing observer performance between WSI and light microscopy for this task [36, 37]. Other validation studies, such as the one by Fine et al. [38], reported on the review of IHC slides but that review was one of multiple components to render primary diagnosis and they did not report on the direct comparison of optical and digital modalities for IHC directly.

We chose IHC assessment of tissue-based biomarkers as we wanted to focus on specific, well-defined pathology tasks, with distinctive features and scoring criteria, thus attempting to minimize sources of variability due to observer training and experience that might dilute differences between the two modalities (optical and digital). Moreover, our study included a relatively large number of pathologists (8 total with varying experience) and a controlled pathology review environment. All reviews took place in a single office, using the same microscope, the same computer/color calibrated monitor combination, and the same ambient light, in order to eliminate any effects these parameters could have on the overall assessment. The goal was to compare observer performance between optical and digital microscopy (interobserver agreement for each modality as well as intermodality agreement for each observer) and the effect of the following study factors.

1. The review of whole sections versus the review of predefined field-of-view (FOV): it had been suggested that the activity of searching through a large tissue section in order to find the tumor regions and then integrating multiple fields into a composite score may introduce observer variability [39–41]. In order to explore the effect of searching, we incorporated the review of tissue microarrays (TMAs), [42, 43] as a means to present a predefined FOV, thus reducing the observer search component and the process of combining regions of interest into a composite score.
2. The review of two different biomarkers (two different tasks), one based on membranous staining (HER2) and the other based on nuclear staining (Ki-67): the evaluation of different intercellular staining is based on different features and as such could have an effect on overall assessment [34, 44]. In addition, the interpretation of intensity of staining has proven to be more subjective than evaluating the amount of staining present [34]; thereby the scoring system utilized can also impact the levels of observer agreements. HER2 (human epidermal growth factor receptor 2, or HER2/*neu*) is primarily used to identify likely responders to adjuvant* trastuzumab* therapy (*Herceptin*, Genentech Inc., San Francisco, California, USA) in breast and gastric cancer [45]. Ki-67 has become one of the most widely used methods for determining proliferative rate in tumor samples [46–49]. In addition to their clinical significance, these two antibodies were selected because they target different cellular locations (HER2 targets membranous staining, whereas Ki-67 targets nuclear staining) and have well defined staining protocols and scoring systems.
3. The collection and analysis of both continuous and categorical data (scores of biomarker expression) to investigate whether they had an effect of overall agreement.

To the best of our knowledge, this is the first observer-based validation study of DP that examined the above factors while making efforts to standardize the reviewing environment.

## 2. Materials and Methods

### 2.1. Whole Slide and TMA Construction

The materials included in the reader study consisted of 25 whole slides stained with HER2, 25 slides stained with Ki-67, 1 TMA consisting of 97 0.6 mm cores stained with HER2, and 1 TMA consisting of 97 0.6 mm cores stained with Ki-67. Whole slides and TMAs were constructed from 25 formalin fixed, paraffin embedded breast cancer specimens obtained from the Tissue Array Research Program (TARP). From each patient block three slides were generated and stained with HER2, Ki-67, and hematoxylin and eosin (H&E). Areas of tumor were carefully selected by a pathologist (Stephen M. Hewitt) from the H&E slides using optical microscopy. Between two and eight, 0.6 mm diameter cores were retrieved from the donor blocks and placed into a recipient block utilizing a manual TMA arrayer (Beecher Instruments, Silver Spring, MD, USA) as outlined by Kononen et al. [43]. Whole sections and TMAs were sectioned at 4 *μ*m. Inclusion of the biospecimens was approved by the NIH Office of Human Subjects Research. Readers could defer a score if they judged the sample inadequate. Any cases which were deferred by the majority of pathologists were removed from analysis.

### 2.2. Whole Slide and TMA Immunohistochemical Staining

Tissue sections were deparaffinized and hydrated in xylene and serial alcohol solutions, respectively. Antigen retrieval was performed in a steam and pressure cooker with prewarmed antigen retrieval buffer pH 6 and pH 9 (Dako, Carpinteria, CA, USA) at 95°C, for 40 min and 20 min for HER2 and Ki67, respectively. Endogenous peroxidase was blocked by incubation in 3% H_2_O_2_ for 10 min. After washing with TBST, the specimen was incubated with monoclonal mouse anti-human Ki-67 antibodies (Clone MIB-1 Dako, Carpinteria, CA, USA, dilution 1 : 500 60 min, RT), or polyclonal rabbit anti-human HER2 antibody (c-erbB2, catalog number K5204, rabbit polyclonal: Dako, dilution 1 : 500, 30 min, RT). Antigen-antibody reactions were detected with Envision+ peroxidase kit (Dako, Carpinteria, CA, USA). The stain was visualized using 3,3′-diaminobenzidine plus (Dako, Carpinteria, CA, USA) and was counterstained with hematoxylin, dehydrated in ethanol, cleared in xylene, and cover-slipped. Appropriate negative controls were concurrently performed, and the TMAs included appropriate control tissue.

### 2.3. Microscope, Digitization, and Reviewing Software

**Figure 1:**
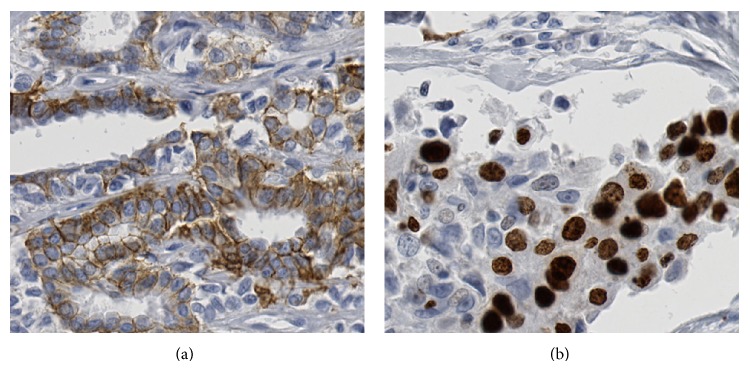
Example of immunohistochemistry for HER2 whole slide (a) and Ki-67 whole slide (b).

**Figure 2:**
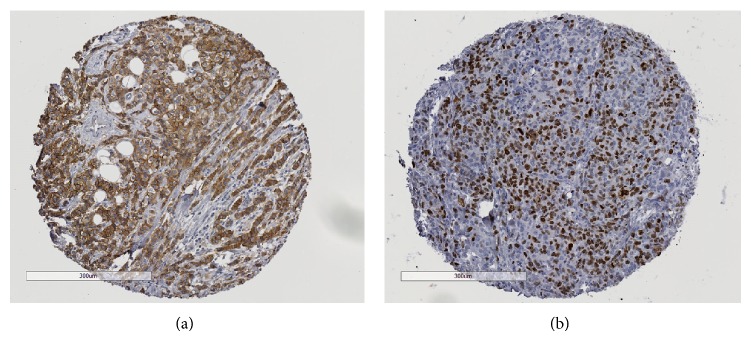
Examples of immunohistochemistry for HER2 TMA core (a) and Ki-67 TMA core (b).

Microscope slides were reviewed using an Olympus BH-2 microscope (Olympus, Center Valley, Pennsylvania, USA), which was drawn from the clinical inventory of the Laboratory of Pathology. Pathologists were able to pan the microscope slide and change the objectives (4x, 10x, 20x, and 40x) without restrictions. All slides were digitized using a Hamamatsu NanoZoomer 2.0 HT (Hamamatsu Photonics, Bridgewater, NY, USA) instrument at the 40x mode (0.23 *μ*m/pixel). Sample regions of interest from whole slides and TMA are shown in Figures 1 and 2, respectively. Digital Images were displayed on a Samsung 27′′ 6 series 1080p LED monitor (Samsung Electronics America, Ridgefield Park, NY, USA). The monitor was calibrated using an Eye-One calibration kit (X-Rite, Tewksbury, MA, USA). Distiller (SlidePath, Dublin, Ireland), a web-based software solution, was used to display the digital images. Within the software, users were able to pan around the image and view the slide at multiple magnifications (4x up to 40x). Users had a choice of keyboard, mouse or trackball navigation, and could switch actively between them. TMA workflow (sequence of cores viewed) was also facilitated within Distiller, thereby eliminating the risk of reporting error when performing the digital TMA review.

### 2.4. Observer Training and IHC Scoring System

All observers were trained using a two-step procedure. First, a PowerPoint presentation was used to describe the objectives of the study, to clarify the details involved with the assessment of HER2 and Ki-67, and to provide guidelines regarding the scoring system. The presentation included reviewing 20 regions of interest. Second, a practice session within the digital viewing software was provided, so that pathologists would become familiar with the controls and digital interface. A digitized TMA unrelated to this study was used for the practice session. The 2-step training was provided at the beginning of each reader session.

The scoring system utilized to evaluate HER2 involved an ordinal system {0, 1 + , 2 + , and  3+} in accordance with the FDA-approved Dako Herceptest scoring system (Dako Inc., Carpinteria, CA, USA). In addition, a “continuous” score of integers between 0 and 100 was utilized. Continuous scores were recorded for the purpose of improving the information content of this study and to examine the effect of using different data types (categorical versus continuous) on performance evaluation. A higher number on the continuous scale represented increasing membrane staining intensity and staining completeness. Users were instructed that continuous scores between 0 and 24 corresponded to the categorical score of 0, continuous scores between 25 and 49 corresponded to the categorical score of 1+, continuous scores between 50 and 74 corresponded to the 2+ category, and continuous scores ≥75 corresponded to the 3+ category. In the study, users (observers) were asked to first provide a categorical a score and then a continuous score.

For Ki-67 interpretation, users first had to evaluate if there were ≥500 and ≥100 tumor cells, for the whole sections and each TMA core, respectively. Only scores for which there was a sufficient number of tumor cells were included in the subsequent analyses. Then they were instructed to provide a positive score if >10% of cells were considered positive for Ki-67 expression, and a negative score otherwise [48]. The interpretation of Ki-67 does not include the classification of intensity of staining, but rather the percentage of tumor cells with positive staining. Additionally, pathologists were asked to provide a “continuous” score in the range of 0 to 100 corresponding to the percentage of positive tumor cells.

Readers could defer a score if they judged the sample inadequate. Cases for which the majority of pathologists deferred to score were removed from analysis. Instances of deferrals in the remaining cases were considered as missing data and were not included in the statistical analysis (not counted in calculating the average pair-wise agreement metrics described below).

### 2.5. Study Design

Eight observers (four anatomic pathology (AP) or anatomic pathology/clinical pathology (AP/CP) board-certified pathologists and four AP residents) reviewed cases in four sessions. There was a minimum washout period of at least 2 weeks between sessions; however the average washout time was about 6 weeks. The sessions were (1) whole slides with optical microscopy (review of all 25 HER2 followed by 25 Ki-67 slides or vice versa), (2) whole slides with digital environment (review of all 25 HER2 followed by 25 Ki-67 slides or vice versa), (3) TMA with optical microscope (review of the HER2 TMA slide followed by reviewing the Ki-67 TMA slide, or vice versa), and (4) TMA with digital environment (review of the HER2 TMA slide followed by reviewing the Ki-67 TMA slide, or vice versa). The order of each session was randomized for each observer, with each whole slide session to be followed by a TMA session to reduce the chance for recall of cases. For both modalities (optical and digital) and biomarkers (HER2 and Ki-67) the order in which the cores were reviewed within the TMA was identical. The order of which biomarker to review first (HER2 or Ki-67) was also randomized for each observer. The overall study duration was approximately 1 year.

### 2.6. Statistical Analysis

The scores from the observer study were analyzed using agreement analysis since IHC interpretation is a subjective method of evaluation with only a semiquantitative scoring system available, at best. Therefore, a definitive truth score for IHC protein expression of HER2 or Ki-67 is not readily available. Besides, agreement between digital and optical scores was the primary objective of the study since microscope-based assessment is primarily considered as the reference standard for IHC assessment. Two well-known measures of agreement were utilized, Kendall's tau-*b* and* percent correct agreement*.

Kendall's tau-*b* is a rank-based correlation metric which calculates the difference between the rate of concordance and discordance while correcting for ties [50]. (Two readers are concordant on a pair of cases if they rank them in the same order.) The range of Kendall's tau-*b* is (−1 to 1), where 1 indicates the readers are always concordant (perfect agreement), −1 indicates they are always discordant (perfect disagreement), and 0 indicates no agreement. Kendall's tau-*b* was computed based on the definition outlined by Woolson and Clarke [51]. Both the continuous and categorical scores were analyzed with Kendall's tau-*b*, with the exception of the categorical scores for Ki-67, where Kendall's tau-*b* would be ill-defined due the large number of ties.

The second figure of merit used in our analyses was percent correct agreement which was further broken down into (a) overall percent correct agreement,* defined as the percentage of cases for which the scores from two distributions (the scores from 2 observers) coincided*, and (b) category-specific correct agreement (for 0, 1+, 2+, and 3+),* defined as the number of cases for which a score for a specific category was observed in both distributions divided by the number of cases with a score in that category in at least one distribution*.

Kendall's tau-*b* values and percent correct agreement were utilized to quantify* interobserver agreement* (agreement between a pair of pathologists reviewing the same data with the same modality, averaged overall all pairs of pathologists) and* intermodality agreement* (agreement between the scores of the same observer using digital and optical microscopy, averaged over all observers). Confidence intervals for the overall agreement measures were calculated using bootstrap analysis using a procedure described in detail in the study by Gavrielides et al. [40]. This analysis accounts for the variability from cases and readers. All software was implemented using MATLAB (MathWorks, Natick, Massachusetts) functions.

## 3. Results

### 3.1. Excluded Cases and Deferred Scores

**Figure 3:**
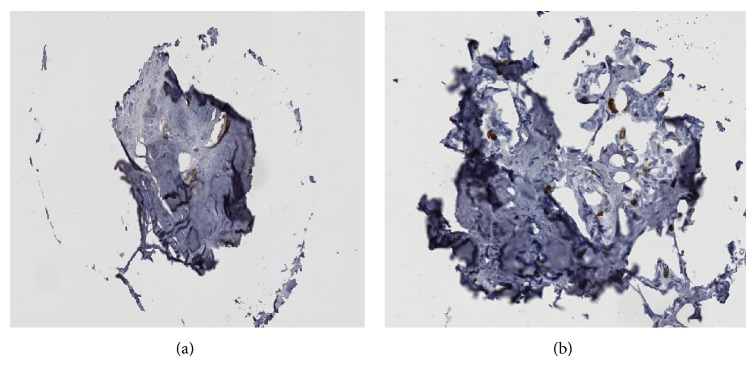
Examples of a deferred TMA cores stained with HER2 (a) and Ki-67 (b).

For the HER2 and Ki-67 TMAs, 9 and 8 cores (out of 97), respectively, were deferred by the majority of the pathologists due to either poorly prepared tissue or not enough tumor tissue and were excluded from further analysis.  Figure 3 shows examples of excluded TMA cores stained with HER2 and Ki-67, respectively. From the review of the remaining 88 HER2 cores, 13 out 704 possible scores (8 pathologists × 88 cores) were deferred with optical and 10 out of 704 were deferred with digital microscopy. From the review of the 89 Ki-67 remaining cores, 120 out of 712 possible scores (8 pathologists × 89 cores) were deferred with optical and 103 out of 712 were deferred with digital microscopy. For HER2 whole slides, 1 out of 200 possible scores was deferred using optical microscopy (none with digital). For Ki-67 whole slides, 9 out of 200 possible scores were deferred using optical microscopy, 7 out of 200 with digital. All nondeferred scores were used in the following analyses.

### 3.2. Interobserver Variability for the Assessment of HER2

Table 1 shows Kendall's tau-*b* values for overall interobserver agreement in the assessment of HER2 on whole slides and on TMA. Our primary comparison shows a nonsignificant trend for better agreement with optical readings of whole slides and no difference in agreement for the different viewing modes of the TMA. Agreement was relatively high overall, ranging from 0.67 to 0.75 for readings of whole slides and values of 0.80 for reads on the TMA. Better agreement for the TMA is perhaps due to the reduced need to search and aggregate a score across different fields. This finding needs to be tested further as we are unable to claim statistical significance for the difference in agreement. For whole slides, the 95% confidence intervals (95% CIs) were quite large due to the moderate number of observations (25 slides), whereas the 95% CIs for TMAs were tighter thanks to the larger number of observations (88 for HER2, 89 for Ki-67).

Table 2 shows overall interobserver percent correct agreement in the assessment of HER2 on whole slides and on TMA, as well as category-specific percent correct agreement. The same patterns are replicated as with Kendall's tau-*b*, of (a) better agreement on optical versus digital read on whole slides and less significant differences on the TMA, (b) slightly better agreement on TMAs compared with whole slides, and (c) tighter 95% CIs for TMAs. As expected, results showed higher percent correct agreement for the 0 and 3+ categories compared to the lower agreement for the 1+ and 2+ categories.

### 3.3. Intermodality Variability in the Assessment of HER2

Table 3 tabulates Kendall's tau-*b* values for overall intermodality agreement for the assessment of HER2. Generally, intermodality values appear to be comparable to the interobserver values. The same pattern of greater agreement when using TMAs compared to whole sections is also evident.  Table 4 shows overall percent correct agreement, as well as category-specific percent correct agreement. Results show again greater intermodality agreement in the review of TMAs, much tighter 95% CIs for TMAs, and higher percent correct agreement for the 0 and 3+ categories compared to the agreement for the 1+ and 2+ categories.

### 3.4. Interobserver Variability for the Assessment of Ki-67

Results for the analysis of interobserver variability for the assessment of Ki-67 are shown in Tables 5 and 6. Overall interobserver agreement with Kendall's tau-*b* was again relatively high, ranging within 0.75-0.76 on the whole slides and 0.71–0.74 for reads on the TMA. The trend observed here is opposite to the one observed with HER2, with slightly better concordance on whole sections rather than TMAs. Additionally, unlike the case for HER2, our primary comparison shows optical and digital reads are at almost identical levels, independent of the format (whole slides versus TMA). Results show again much tighter 95% CIs for TMAs.

Percent correct results for the Ki-67 binary data (Table 6) were not consistent with Kendall's tau-*b* results given the continuous data. Agreement on whole slides with digital was higher than with optical (again, no statistical significance) for categorical scores (positive versus negative) whereas it was practically equal for continuous scores. We lack a concrete explanation for this result, except saying that the error bars are generous enough to allow for such inconsistencies and that differences between observer scoring for continuous and binary could be due to possible unfamiliarity with the use of the continuous scale for Ki-67 (or counting tasks in general) despite our training process. Regarding our primary comparison, there is no evidence that either optical or digital reading is superior. Likewise, there is no trend regarding the impact that evaluation area has on agreement.

Overall, despite ambiguous results, especially regarding the different scoring methods (continuous versus binary), interobserver agreement for both whole slides and the TMA was at a similar level for the two viewing modes in the assessment of Ki-67.

### 3.5. Intermodality Variability in the Assessment of Ki-67

Table 7 shows Kendall's tau-*b* values quantifying intermodality agreement in the assessment of Ki-67. Results for whole slides show comparable intermodality agreement values to those derived from interobserver agreement analysis. For TMA assessment, intermodality agreement is moderately improved compared to interobserver agreement. As previously observed, the 95% CIs are tighter for TMA review in this analysis. Looking at percent correct values (Table 8), intermodality agreement was at relatively high values and at comparable levels to interobserver agreement for whole slide assessment.

## 4. Discussion

Digital pathology is an emerging field that is becoming more commonplace in routine pathology practice. The U.S. Food and Drug Administration has cleared a number of 510k applications for devices to quantify IHC expression for HER2 (available on FDA 510k database) and a number of other tissue-based biomarkers as an aid in diagnosis (ER and PR), as well for the assessment of HER2/neu for digital manual read in the USA. The FDA convened a public panel in 2009 to address regulatory issues for digital pathology and currently considers digital pathology devices with an intended use of rendering a* primary* diagnosis as Class III devices, requiring validation studies to ensure safety and effectiveness. The comparison study in this paper evaluates only a small aspect of the issues related to determining primary diagnoses from computer screens, focusing on observer variability in IHC stain evaluation, for specific IHC tasks, performed on whole slides and TMAs and analyzed using continuous and categorical data, and different agreement metrics. We collected paired observations while accounting for possible sources of interobserver and intermodality variability by using a common clinical microscope, digital environment, ambient conditions, and observer training. The objective was clearly not to recreate the typical clinical work flow; issues related to workflow such as comparing time taken for IHC evaluation between WSI and digital were not examined in this study.

The results of the study show relatively high overall* intermodality* agreement (optical versus digital) for IHC assessment with values depending on which biomarker was reviewed and how it was reviewed. For HER2, agreement quantified using Kendall's tau-*b* on continuous data ranged from 0.73 (whole slides) to 0.83 (TMA) for the samples used. In a previous study by this group, Kendall's tau-*b* for interobserver agreement for HER2 in an observer study of 241 regions of interest read by 7 pathologists was 0.61 (95% CI: 0.53–0.67) [40]. Results also show relatively high* interobserver agreement* for IHC assessment with either digital or optical microscopy with values again depending on which biomarker was reviewed and how it was reviewed. For Ki-67, intermodality and interobserver agreement were also high, with Kendall's tau-*b* values on continuous data higher than 0.71.

Despite these relatively high agreement values, our findings indicate that significant interobserver variability exists for IHC tasks. That was especially evident for the 1+ and 2+ categories of HER2 for which agreement was relatively low. An analysis of intermodality disagreement in our study showed that on the average 28% of the TMA cores scored as 1+ with optical microscopy were scored as 2+ with digital by the same observer, and 25% of TMA cores scored as 2+ with optical microscopy were scored as 3+ with digital. Such disagreements could have clinical significance, since they could possibly lead to unnecessary follow-up testing (in the case of 1+ scored as 2+), or false-positives subject to unnecessary treatment with related side effects (in the case of 2+ scored as 3+). Similarly, interobserver analysis showed that on the average 26% of the TMA cores scored as 1+ with digital microscopy by one observer were scored as 2+ by another observer, and 13% of the whole slides scored as 2+ with optical microscopy were scored as 3+ with digital (interestingly, 28% of the 2+ were scored as 1+ unlike the case for intramodality analysis where most disagreement was on the side of 3+). Comparable results were derived for interobserver analysis on optical microscopy. These findings support the use of computer aids for IHC tasks that were shown to improve inter- and intraobserver agreement [40].

Our study raised questions about the interaction of tasks with scoring methods. While for HER2 results were similar in terms of agreement patterns between the analysis of continuous scores and categorical scores, as seen through different agreement measures (Kendall's tau-*b* and PA), some different patterns were seen between the analyses for Ki-67. These patterns are not definitive in a statistical sense. However, we feel that they point to differences in scoring methods and the associated training and experience with a task. For HER2, the scoring of membrane staining into four HER2 categories (3+ cases corresponded to continuous scores of 75–100, 2+ corresponded to continuous scores of 50–74, and so on) is a well-established process, used in clinical practice, and one that pathologists are familiar with. In contrast, Ki-67 scoring involved estimation of the percentage of nuclear-stained cells. Although based on clinical practice, the evaluation of Ki-67 as positive or negative based on an estimation of greater or less than 10% of tumor cells expressing the marker is not as established or commonly encountered and incorporates a determination of a threshold of staining to be considered positive. Individual observers often use different strategies to sample cells for counting and calibrating their continuous scores might not be as straightforward as for HER2.

Similarly, in terms of comparing the review of whole slides and TMA, there were again different trends observed for the two biomarkers; for HER2 interobserver and intermodality agreement were higher for TMA compared to whole slides, suggesting a possible benefit in a restricted field of view, whereas for Ki-67 agreement was practically equivalent.

Recent validation studies for primary diagnosis use the “broader scope” approach [52–54], where diagnoses on multiple tissue types from multiple organs and using multiple stains are compared between optical and digital microscopy. Such studies are valuable in examining clinical workflow and operational challenges as discussed in a recent editorial article [54]. However, pooling cases with different diagnoses from different tissue types may dilute differences in observer patterns and biases for specific tasks and mask important features and limitations of digital pathology. Additionally, pooling cases with different diagnoses from different tissue types presents a sample size issue. Considering the large number of combined diagnoses and different tissue types in pathology, even for a relatively large study of 607 slides as in the recent study by Bauer et al. [52], a specific individual task is represented by only a few cases. As such, case variability and the interaction of observers with different cases for individual tasks might not be captured in studies designed with the broader scope approach. In our study, despite very limited and specific tasks with immunohistochemistry assessment that is typically simpler than primary diagnosis, wide confidence intervals were observed in our analysis of 25 whole slides: 95% CIs ranged from 0.49 to 0.80 and from 0.61 to 0.86 for the assessment of HER2 and Ki-67, respectively, with digital microscopy. We observed these high levels of uncertainty even though we controlled for study variables like the choice of stain and scoring method, the use of a common microscope, a common color managed digital environment, and common training and instructions for all observers. In contrast, we saw in our study that for the TMA task where 8 readers read approximately 90 cores per TMA, the confidence intervals were acceptable (width of about 0.10 to 0.15).

Our results support the need for validation studies with* adequate sampling per task*. Such studies would help define the role of digital pathology by determining the clinical tasks for which it can safely and effectively replace the microscope, and they can identify areas where digital pathology technology can be improved. Regardless of standardized protocols that are or will become available, such studies might be needed within each laboratory and by their own pathologists prior to converting to digital pathology for a specific procedure.

In summary, our study demonstrated comparable interobserver agreement in the quantitative assessment of HER2 and Ki-67 for breast cancer with optical and digital microscopy, as well as relatively high intermodality agreement, supporting the potential of digital microscopy for these tasks. Our results identified differences in agreement patterns when examining different biomarkers, different scoring methods, and different fields-of-view, stressing the need for validation studies focused on specific tasks and study designs to eliminate a possible contribution of such differences to the overall observer variability. Finally, the statistical uncertainty observed in our study, even after attempting to minimize such sources of variability, calls for adequate sampling for each individual task rather than pooling cases from different intended tasks.

## Figures and Tables

**Table 1:** Comparison of overall interobserver agreement for the assessment of HER2 between the optical microscope and digital environment, across TMA and whole slides, using Kendall's tau-*b* metric [95% CI].

Interobserver HER2	Kendall's tau-*b* on continuous scores [95% CI]			Kendall's tau-*b* on categorical scores [95% CI]		
	Optical	Digital	Difference	Optical	Digital	Difference
Whole slides	0.75 [0.58–0.87]	0.67 [0.49–0.80]	0.08 [–0.02–0.19]	0.77 [0.61–0.88]	0.69 [0.49–0.80]	0.09 [–0.03–0.21]
TMA	0.80 [0.71–0.86]	0.80 [0.73–0.85]	0.00 [–0.06–0.05]	0.79 [0.71–0.86]	0.83 [0.73–0.85]	–0.04 [–0.11–0.02]

**Table 2:** Comparison of overall interobserver agreement for the assessment of HER2 between the optical microscope and digital environment, using the percent correct agreement metric, as well as category-specific percent correct agreement [95% CI].

Interobserver HER2	Percent correct		
Scoring category	Optical whole slides	Digital whole slides	Difference

All	65.9 [52.4–78.9]	59.4 [46.5–72.0]	6.4 [−8.5–21.5]
0	65.6 [41.7–93.8]	49.7 [14.3–83.7]	15.9 [−20.8–54.8]
1+	37.7 [13.1–62.0]	29.4 [9.2–52.0]	8.3 [−25.3–39.7]
2+	44.0 [26.5–60.8]	36.2 [18.1–54.2]	7.8 [−18.0–33.8]
3+	66.6 [39.6–88.7]	58.4 [35.4–79.0]	8.2 [−14.0–30.5]

	Optical TMA	Digital TMA	Difference

All	71.7 [64.7–78.2]	72.3 [64.4–79.7]	−0.5 [−8.8–7.8]
0+	65.4 [50.3–78.1]	74.9 [59.3–88.0]	−9.5 [−23.5–4.1]
1+	48.6 [36.9–59.0]	45.3 [31.0–59.7]	3.3 [−12.4–18.5]
2+	38.1 [23.7–52.6]	36.4 [24.6–48.1]	1.8 [−14.5–17.3]
3+	77.7 [61.6–90.3]	79.7 [66.4–90.5]	−2.0 [−17.7–13.7]

**Table 3:** Intermodality (optical versus digital) agreement in the assessment of HER2 with whole slides and TMA using Kendall's tau-*b* metric [95% CI].

Intermodality HER2	Kendall's tau-*b* on continuous scores	Kendall's tau-*b* on categorical scores
Whole slides	0.73 [0.56–0.85]	0.72 [0.54–0.84]
TMA	0.83 [0.76–0.89]	0.83 [0.76–0.88]

**Table 4:** Intermodality agreement (optical versus digital) in the assessment of HER2 with whole slides and TMA using percent correct and category-specific percent correct agreement [95% CI].

Intermodality HER2	Percent correct	
Scoring category	Whole slides	TMA
All	58.8 [45.0–72.5]	75.1 [69.1–80.7]
0	55.6 [21.4–85.7]	72.0 [56.9–83.8]
1+	30.1 [9.4–54.4]	52.0 [39.8–63.2]
2+	38.4 [22.4–53.8]	41.9 [28.1–55.1]
3+	54.8 [26.7–79.0]	77.5 [62.6–88.7]

**Table 5:** Comparison of overall interobserver agreement between the assessment of Ki-67 with optical microscope and digital environment, using Kendall's tau-*b* metric [95% CI].

Interobserver Ki-67	Kendall's tau-*b* on continuous scores		
	Optical	Digital	Difference
Whole slides	0.75 [0.63–0.84]	0.76 [0.61–0.86]	−0.01 [−0.13–0.12]
TMA	0.71 [0.63–0.78]	0.74 [0.65–0.80]	−0.03 [−0.09–0.04]

**Table 6:** Comparison of overall interobserver agreement for the assessment of Ki-67 between the optical microscope and digital environment, using the percent correct agreement metric, as well as category-specific percent correct agreement [95% CI].

Interobserver Ki-67	Percent correct		
Scoring category	Optical whole slides	Digital whole slides	Difference

All	78.3 [66.0–88.5]	85.4 [75.0–94.5]	−7.2 [−19.5–4.5]
0	54.2 [33.0–73.4]	63.7 [37.7–84.6]	−9.5 [−29.9–11.6]
1+	70.7 [53.1–85.1]	80.6 [66.1–92.6]	−9.9 [−25.7–5.4]

	Optical TMA	Digital TMA	Difference

All	82.4 [74.4–89.2]	80.5 [71.0–88.2]	1.9 [−5.4–10.2]
0+	68.3 [54.5–80.0]	65.2 [50.6–78.3]	3.1 [−8.4–15.2]
1+	72.3 [60.5–82.6]	69.5 [55.5–81.3]	2.8 [−7.5–14.1]

**Table 7:** Intermodality agreement (optical versus digital) in the assessment of Ki-67 with whole slides and TMA using Kendall's tau-*b* metric [95% CI].

Intermodality agreement for Ki-67	Kendall's tau-*b* on continuous scores
Whole slides	0.78 [0.67–0.87]
TMA	0.78 [0.71–0.82]

**Table 8:** Intermodality agreement (optical versus digital) in the assessment of Ki-67 with whole slides and TMA using percent correct and category-specific percent correct agreement [95% CI].

Intermodality Ki-67	Percent correct
Scoring category	Whole slides

All	86.4 [76.5–94.5]
0	68.3 [47.3–85.3]
1+	80.6 [65.1–92.5]

	TMA

All	86.8 [80.5–92.2]
0	74.3 [62.1–85.0]
1+	78.1 [66.7–87.4]
